# Awake prone positioning for patients with COVID-19 pneumonia in intensive care unit: A systematic review and meta-analysis

**DOI:** 10.3389/fmed.2022.984446

**Published:** 2022-09-09

**Authors:** Hui-Bin Huang, Yan Yao, Yi-Bing Zhu, Bin Du

**Affiliations:** ^1^Department of Critical Care Medicine, Beijing Tsinghua Changgung Hospital, School of Clinical Medicine, Tsinghua University, Beijing, China; ^2^Department of Emergency, Guang'anmen Hospital, Beijing, China; ^3^Medical ICU, Peking Union Medical College Hospital, Beijing, China

**Keywords:** awake prone positioning, COVID-19, intubation, intensive care unit, mortality

## Abstract

**Background:**

Awake prone positioning (APP) has been widely used in non-intubated COVID-19 patients during the pandemic. However, high-quality evidence to support its use in severe COVID-19 patients in an intensive care unit (ICU) is inadequate. Therefore, we aimed to assess the efficacy and safety of APP for intubation requirements and other important outcomes in this patient population.

**Methods:**

We searched for potentially relevant articles in PubMed, Embase, and the Cochrane database from inception to May 25, 2022. Studies focusing on COVID-19 adults in ICU who received APP compared to controls were included. The primary outcome was the intubation requirement. Secondary outcomes were mortality, ICU stay, and adverse events. Study quality was independently assessed, and we also conducted subgroup analysis, sensitivity analysis, and publication bias to explore the potential influence factors.

**Results:**

Ten randomized controlled trials with 1,686 patients were eligible. The quality of the included studies was low to moderate. Overall, the intubation rate was 35.2% in the included patients. The mean daily APP duration ranged from <6 to 9 h, with poor adherence to APP protocols. When pooling, APP significantly reduced intubation requirement (risk ratio [RR] 0.84; 95%CI, 0.74–0.95; *I*^2^ = 0%, *P* = 0.007). Subgroup analyses confirmed the reduced intubation rates in patients who were older (≥60 years), obese, came from a high mortality risk population (>20%), received HFNC/NIV, had lower SpO_2_/FiO_2_ (<150 mmHg), or undergone longer duration of APP (≥8 h). However, APP showed no beneficial effect on mortality (RR 0.92 [95% CI 0.77–1.10; *I*^2^ = 0%, *P* = 0.37] and length of ICU stay (mean difference = −0.58 days; 95% CI, −2.49 to 1.32; *I*^2^ = 63%; *P* = 0.55).

**Conclusion:**

APP significantly reduced intubation requirements in ICU patients with COVID-19 pneumonia without affecting the outcomes of mortality and ICU stay. Further studies with better APP protocol adherence will be needed to define the subgroup of patients most likely to benefit from this strategy.

## Introduction

The prone positioning (PP) is common respiratory support to improve oxygenation in acute respiratory distress syndrome (ARDS) in the intensive care unit (ICU) (1). Studies have demonstrated that those who benefit more from the PP are mechanically ventilated patients with moderate to severe ARDS and improved mortality (2). Therefore, before the COVID-19 epidemic, this strategy was rarely applied in non-intubated or awake patients with acute respiratory failure. After the outbreak occurred, the escalating number of invasive mechanical ventilation for COVID-19 pneumonia led to ICU overload. Moreover, medical resource limitations (3), intubation complications (4), and the potential risk of infection among medical staff (5) promote clinical exploration of the PP feasibility in awake or non-intubated patients. Thus, awake PP (APP) has been widely used in COVID-19 management, and studies focusing on APP's efficacy, safety, and tolerability in such a patient population continue to emerge (6).

Several meta-analyses focusing on the effects of APP on non-intubated or awake COVID-19 patients have been published (6–9). However, these studies yield different results with significant unexplained heterogeneity. The main reason for this is that these meta-analyses included only a small number of early studies (9), only observational studies (7, 9), or recruited patients from various scenarios (emergency department, general ward, and ICU) (6–9). In a recent, well-designed meta-analysis (8), the authors focused on the high-quality evidence for APP in treating patients with COVID-19 pneumonia from various settings. Their subgroup analysis found APP reduced intubation in ICU patients with COVID-19. However, only three RCTs concerning the ICU patients were included (10–12), and the result was not robust for driven by one large RCT (10). In addition, which subgroup of severe COVID-19 patients admitted to the ICU could benefit more from APP and the appropriate duration of APP for these patients was unclear. This may partly explain why the latest Save Sepsis campaign guideline suggested insufficient evidence to support APP for COVID-19 in non-intubated patients with severe COVID-19 (13).

Several RCTs on this topic have recently been published (14–16). With the power of meta-analysis, we aimed to conduct an updated meta-analysis enrolling only COVID-19 patients in the ICU who received APP. We focused on analyzing the evidence of APP based on RCT studies for risk of intubation and other important clinical outcomes. In addition, we further explored the subgroup of the ICU population that could benefit from APP treatment.

## Method

We performed this systematic review and meta-analysis following the PRISMA statement (17) (Supplementary material 1), and our protocol has been registered on the International Platform of Registered Systematic Review and Meta-analysis Protocols database (Registration number: INPLASY202260002).

### Search strategy

Two authors (H-BH and Y-BZ) independently conducted a computerized search of PubMed, Embase, and the Cochrane Library databases up to May 25, 2022 (the last search) for eligible studies without language limitation. Briefly, search terms included (awake prone positioning AND (critical care OR critically ill OR intensive care) AND COVID-19 OR SARS-CoV-2) using MeSH and keywords. Details in the literature search strategy were presented in Supplementary material 2. We evaluated the reference lists of relevant studies and searched on ClinicalTrial.gov, if required, to ensure the inclusion of all potential studies. For republished studies, we included the latest published or reported more complete data. Disagreements were solved by discussions between the two authors.

### Selection criteria

Studies were considered for eligibility if they fulfilled the following criteria: (1) study should recruit awake or non-intubated adults (>18 years old) requiring ICU admission due to COVID-19 pneumonia; intermediate care unit and any severe COVID-19 patient unit were classified as ICU; (2) study should compare APP (APP group) with supine position (control group); (3) predefined outcomes included intubation rate, mortality or length of stay in ICU; and (4) the study design included RCTs or observational studies (prospective or retrospective design). We excluded studies enrolling pregnant women or patients with pre-existing dementia or brain injury. Articles published in editorials, comments, protocols, case series, and narrative reviews without data on predefined outcomes were also excluded.

After a thorough computerized search, the two authors (H-BH and Y-BZ) independently examined the titles and abstracts and identified potentially suitable papers. When either of the authors considered that the citations might fit the criteria for inclusion, a full-text review was done.

### Data extraction and outcomes

The two authors (H-BH and Y-BZ) collected the associated data independently on the first author's name, year of publication, setting, study design, enrolment location, patient characteristics (age, male percentage, body mass index, and disease severity), APP and control regimens, as well as predefined outcomes.

The primary outcome was the intubation rate in ICU. Secondary outcomes included all-cause mortality at the longest follow-up available, length of stay (LOS) in ICU, oxygenation, and adverse events (as defined by each author). Discrepancies were identified and resolved through discussion.

### Quality assessment

H-BH and Y-Y independently evaluated the methodological quality of the individual studies using the Cochrane risk of bias tool for RCTs (18) and the Newcastle-Ottawa Quality Assessment Scale (19) for case-control and cohort studies. We evaluated publication bias by visually inspecting funnel plots when at least ten studies were included in this meta-analysis. The Grading of Recommendations Assessment, Development and Evaluation (GRADE) method was used to grade the quality or certainty of the outcomes and the strength of recommendations.

### Statistical analysis

The results from all relevant studies were combined to estimate the pooled odds ratio (OR) and associated 95% confidence intervals (CI) for dichotomous outcomes (i.e., intubation risk, all-cause mortality, and advert events). As to the continuous outcomes (ICU LOS), we estimated mean differences (MD) and 95% CI as effective results. For studies that reported median with an accompanying interquartile range (IQR) as the measure of treatment effect, we estimated the mean from median and standard deviations (SD) from IQR using the methods described in previous studies before data analysis (20). We selected the results from intention-to-treat rather than per-protocol or as-treated if required.

In analyzing each predefined outcome, we conducted meta-analyses separately on RCTs and observational studies, while the results of observational studies were only presented in the Additional file. To test the robustness of the outcomes and explore the potential influence factors, we conducted sensitivity analyses to investigate the influence of a single study on the overall pooled estimate of each predefined outcome. Specifically, we conducted sensitivity analyses of HFNC+APP vs. HFNC alone and NIV+APP vs. NIV alone. Additionally, subgroup analysis was performed separately by pooling studies basing on (1) sample size: ≥200 or <200; (2) high flow nasal cannula (HFNC)/non-invasive ventilation (NIV) percentage: ≥50 or <50%; (3) mean SaO_2_/FiO_2_: ≥150 or <150 mmHg; (4) actual daily APP duration: ≥8 or <8 h; (5) obesity percentage: ≥40 or <40%; (6) mortality prevalence: ≥20 or <20%, and (7) age: ≥60 or <60 for all the outcomes of interest.

We used the *I*^2^ statistic to test the heterogeneity (21). An *I*^2^ < 50% was considered as insignificant heterogeneity, and a fixed-effect model was used, whereas a random-effect model was used in cases of significant heterogeneity (*I*^2^ > 50%) using the Mantel-Haenszel method. The threshold for significance for *P* values was 0.05. We performed all analyses using Review Manager, Version 5.4.

## Results

### Searching results

**Figure 1:**
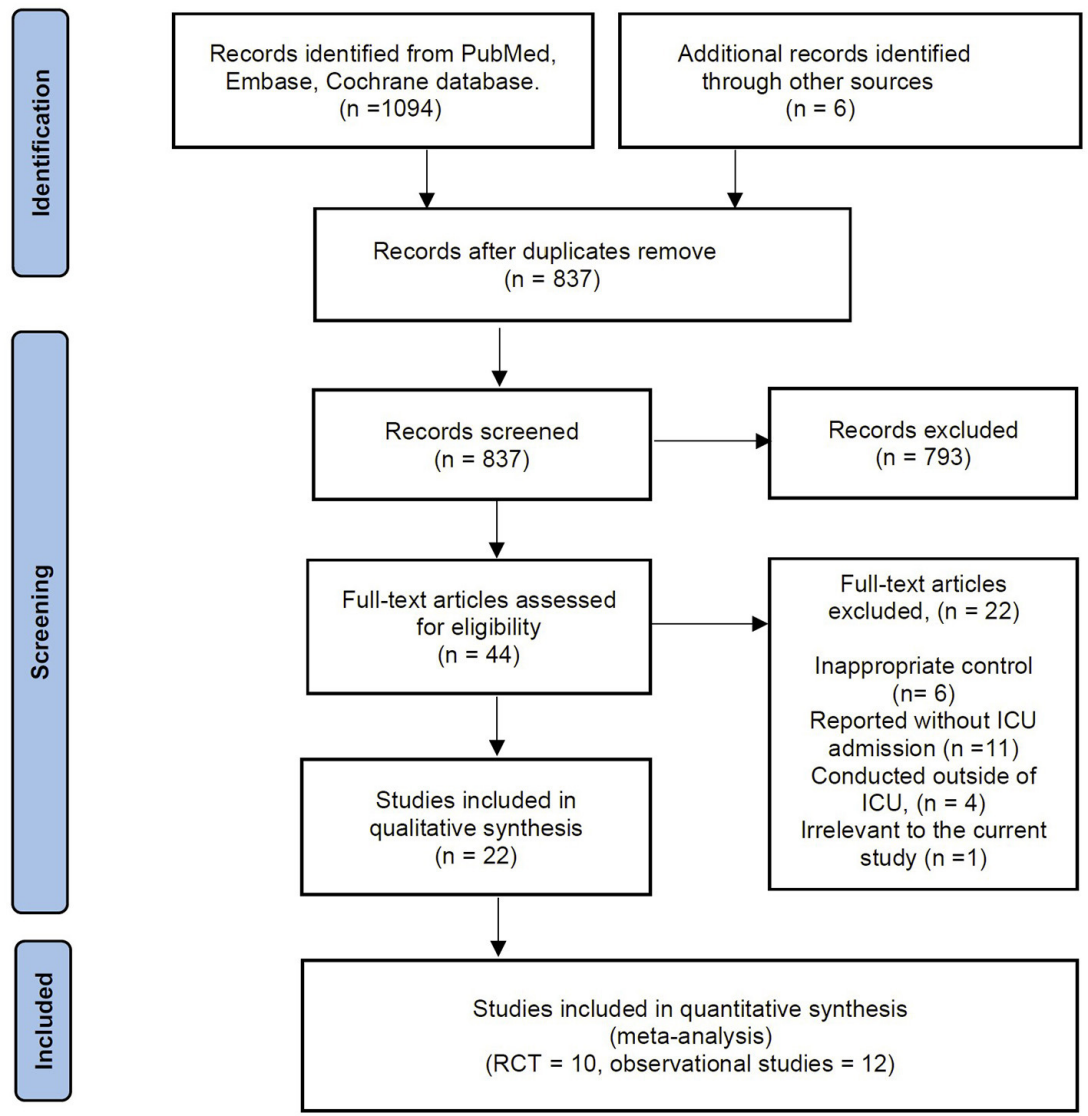
Selection process for the studies included in the meta-analysis.

The electronic search yielded 1,094 records from the databases, and another source produced six records. There were 837 records after de-duplications, of which we excluded 873 records based on title and abstract screening. A total of 44 studies were considered for full-text review. After a full-text review, we excluded 22 articles summarized in Supplementary material 3 for exclusion reasons. Thus, 5 RCTs with 1,686 patients and 12 observational studies with 1,522 patients were potentially eligible for inclusion. However, of the 5 RCTs (10–12, 14, 16), the study by Ehrmann et al. (10) comprises six independent registered trials at ClinicalTrials.gov [NCT04347941 (22), NCT04325906 (23), NCT04358939 (24), NCT04391140 (25), NCT04395144 (26), and NCT04477655 (27)]. Considering the potentially considerable heterogeneity between the six trials, we included these trials independently in our meta-analysis. Moreover, we found the newly published RCT by Ibarra-Estrada and colleagues (15) was conducted on the same cohort as the registered trial of NCT04477655 (27) while providing more associated data. Therefore, this RCT was selected in the current meta-analysis (Figure 1). Finally, we included these ten RCTs in our analysis (11, 12, 14–16, 22–26). In addition, the details of the reference list for the 12 included observational studies are available in Supplementary material 4.

### Study characteristics and quality assessment

The main characteristics and the respiratory therapy regimens of the ten RCTs are shown in Tables 1, 2. These studies were conducted between 2020 and 2022, with the sample size ranging from 13 to 430. Then, 850 patients were analyzed in the APP group and 836 in the control group. All but one (11) of these trials were multi-center RCTs. Different types of initial respiratory support were used among the included studies, of which HFNC was the most used (*n* = 9), and followed by NIV (*n* = 3). Four RCTs set specific targets for daily APP duration (11, 12, 14, 16), while the remaining six encouraged patients to implement APP for as long as they could tolerate (15, 22–26). However, the actual daily APP duration was much lower than expected and varied significantly across the included trials (from 1.6 to 9.0 h/day) (Table 2). The details in characteristics of the included observational studies are available in Supplementary material 4.

**Table 1:** Characteristics of included studies in the current meta-analysis and systemic review.

**Study**	**Country**	**N**	**Design**	**Setting**	**Age, year**	**Male, %**	**BMI, kg/m^2^**	**Obesity, %**	**Follow-up**	**Mortality, %**	**Intubation,%**
Alhazzani et al. (14)	Canada	400	MC	ICU	57/58	73/69	29.7/29.5	NA	60 d	23	34.1/40.5
NCT04325906 (23)	USA	222	MC	ICU+I-CU	62/61	67/66	29.7/29.7	53/56	28 d	22.9	14.3/16.7
NCT04395144 (26)	Canada	13	MC	ICU+I-CU	65/68	57/33	27.4/30.7	17/50	28 d	30.8	38/40.6
NCT04358939 (24)	France	402	MC	ICU	64/63	75/75	28.7/28.9	31/37	28 d	10.2	0/16.7
NCT04347941 (22)	Ireland	24	MC	ICU+I-CU	63/59	75/58	32.2/34.2	50/67	28 d	0	33.9/35.5
NCT04391140 (25)	Spain	30	MC	ICU	58/52	76/77	30.1/28.9	47/39	28 d	10	29.4/53.8
Gad et al. (11)	Egypt	30	SC	ICU	49/46	60/53	NA	33/20	H-LOS	20	20/20
Ibarra-Estrada et al. (15)	Mexico	430	MC	ICU+I-CU	59/58	61/59	30.3/30	40/38	28 d	34.9	30/43
Jayakumar et al. (12)	India	60	MC	ICU	55/57	83/83	28.2/25.8	NA	ICU-LOS	6.7	13.3/13.3
Rosén et al. (16)	Sweden	75	MC	ICU+W	66/65	64/82	28/29	23/32	30 d	12	33.3/33.3

APP, awake prone positioning; BMI, body mass index; HFNC, high-flow nasal cannula; ICU, intensive care unit; I-CU, intermediate care unit; NIV, non-invasive ventilation.

**Table 2:** Respiratory characteristics and treatment regimens in the included patients.

**Study**	**Mean P/F**	**Mean S/F**	**Usual care**	**Targeted daily APP duration**	**Actual daily APP duration, h**	**APP duration in control, h**
Alhazzani 2022 (14)	NA	132/136	HFNC, NIV, LF	8 h/d to 10 h/d with 2 to 3 breaks	5.0 [2.6–8.0]	0 (0–0)
NCT04325906 (23)	NA	152/156	HFNC	As long and as frequently as possible	2·5 [0·7; 6·9]	0·7 ± 2·0
NCT04395144 (26)	NA	169/167	HFNC	As long and as frequently as possible	2·4 [1·7; 3·0]	0 ± 0
NCT04358939 (24)	NA	155/156	HFNC	As long and as frequently as possible	2·0 [1·0; 3·7]	0 ± 0·3
NCT04347941 (22)	NA	194/178	HFNC	As long and as frequently as possible	3·1 [2·1; 3·9]	1·0 ± 2·5
NCT04391140 (25)	NA	163/156	HFNC	As long and as frequently as possible	1·6 [1·1; 2·3]	0 ± 0
Gad et al. (11)	126/111	NA	NRM	1–2 h each session, 3 h apart when awake	<6	NA
Ibarra-Estrada et al. (15)	NA	135/136	HFNC	As long and as frequently as possible	8·6 [6·1; 11·4]	0·3 ± 1·0
Jayakumar et al. (12)	201/186	NA	NC, FM, HFNC, NIV	At least 6 hours a day	<6	NA
Rosén et al. (16)	116/116	151/157	HFNF/NIV	At least 16 hours per day	9.0 [4.4–10.6]	3.4 [1.8–8.4]

Data was presented as mean ± SD or median (IQR) or awake prone positioning/Control. APP, awake prone positioning; BMI, body mass index; h, hour; HFNC, high flow nasal cannula; IMV, invasive mechanical ventilation; NA, not available; NC, nasal cannula; NIV, non-invasive ventilation; NRM, non-rebreather mask; P/F, ratio of partial pressure of arterial oxygen to fraction of inhaled oxygen; S/F, ratio of pulse oxygen saturation to fraction of inhaled oxygen; SpO2, pulse oxygen saturation.

**Figure 2:**
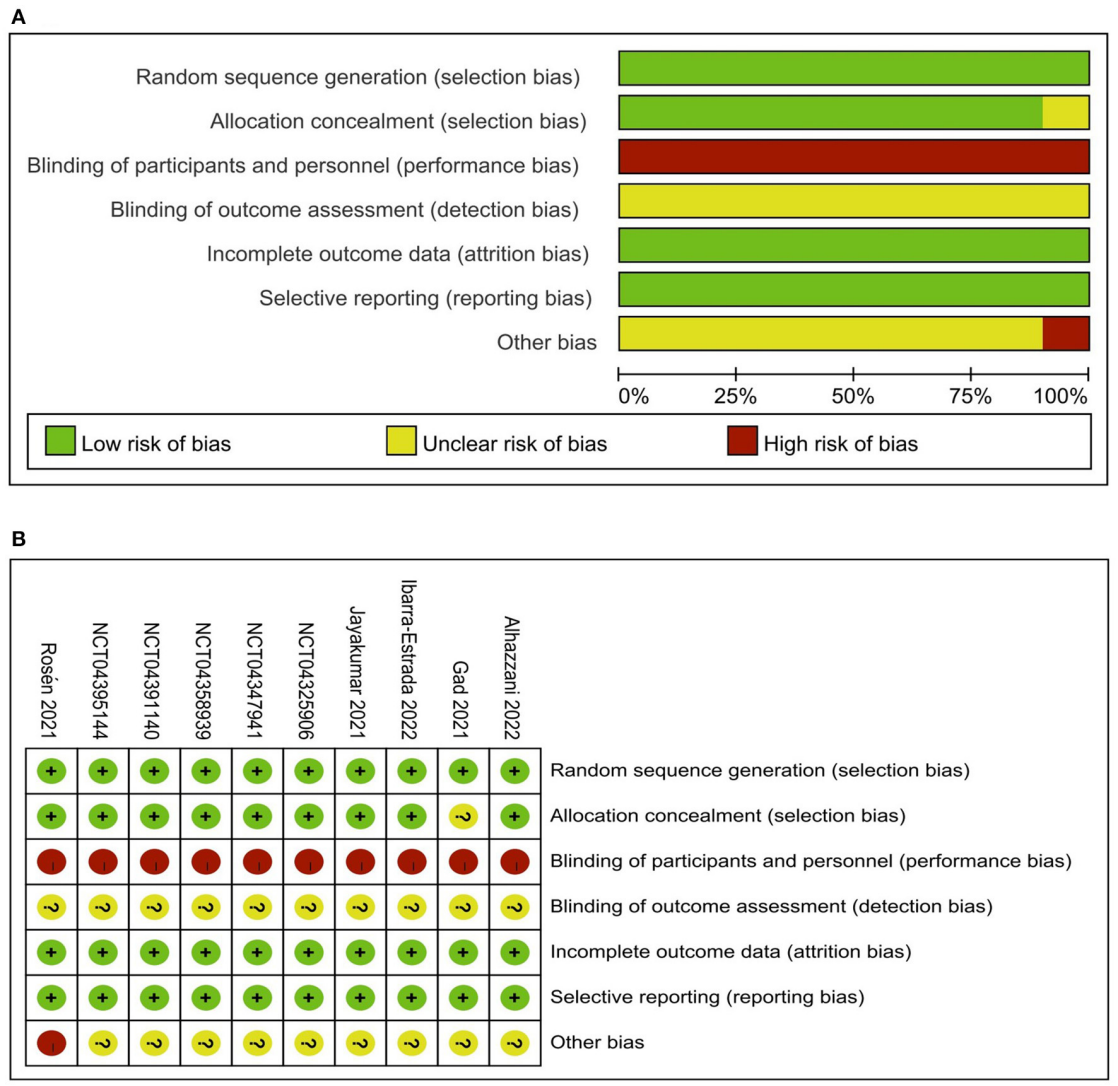
Risk of bias graph: review authors' judgements about each risk of bias item presented as percentages across all included studies **(A)** and Risk of bias summary: review authors' judgements about each risk of bias item for each included study **(B)**.

We evaluated the included studies' risk of bias using the Cochrane risk-of-bias tool for the ten RCTs (Supplementary material 5) and the Newcastle-Ottawa Quality Assessment Scale for the 12 observational studies (Supplementary material 6). The quality of observational studies was moderate to high, and the risk of bias in RCTs was low in all critical domains Figure 2. Assessment of publication bias using visually inspecting funnel plots showed no potential publication bias among the included studies (Supplementary material 7). Using GRADE methodology, we assessed the evidence for pooled data for intubation rate, mortality, and ICU length of stay to be moderate, moderate, and very low, respectively (Supplementary material 8).

### Primary outcome

**Figure 3:**
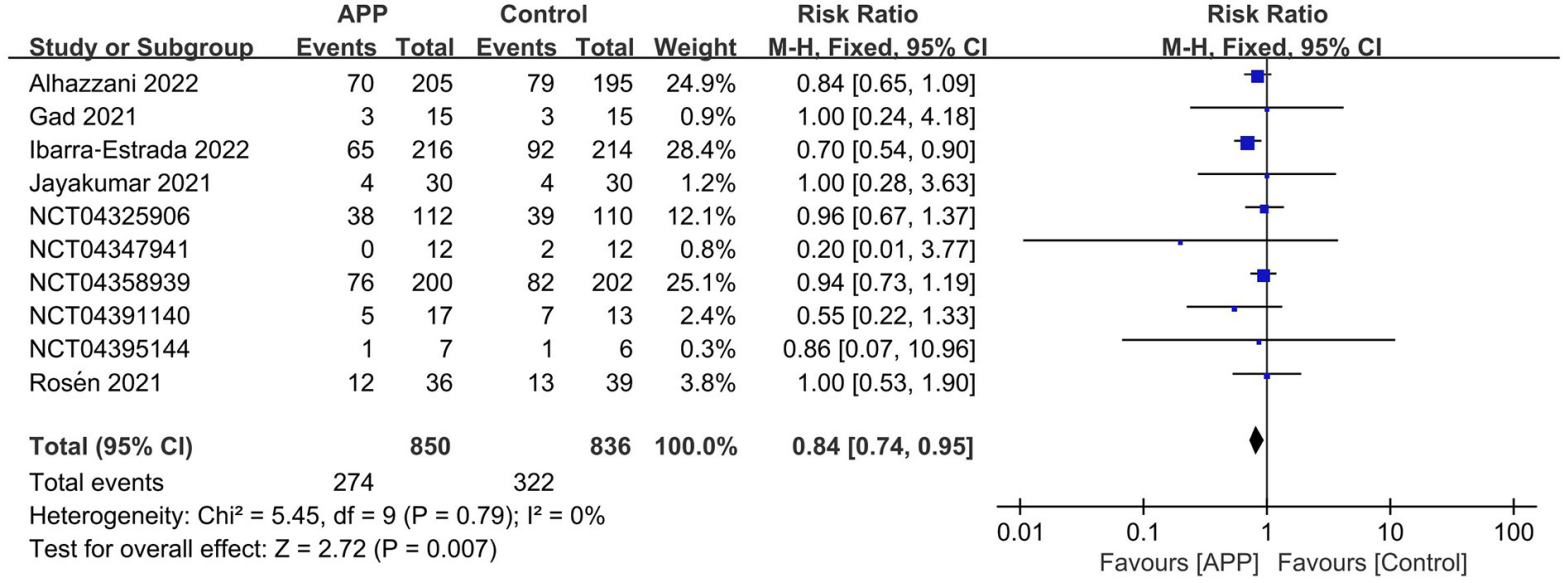
Forest plots of the awake prone position on intubation rates in COVID-19 patients in the intensive care unit.

The outcome of intubation risk was available in all the RCTs (11, 12, 14–16, 22–26). Among these patients, 850 received APP, and 274 were intubated (32.2%) compared to 836 patients of control, with 322 intubated (38.5%) observed. We found that patients with APP significantly reduced the intubation requirement than those in the control group (*n* = 1,686; RR = 0.84; 95%CI, 0.74 to 0.95; *I*^2^ = 0%, *P* = 0.007) (Figure 3). In the sensitivity analysis, excluding any single study did not significantly alter the overall combined RR (*P*-value ranging from 0.0002 to *P* = 0.007). Meanwhile, pooling only RCTs of comparing HFNC+APP vs. HFNC alone showed significant benefits of APP in the outcome of intubation rate (7 RCTs, RR = 0.79; 95%CI, 0.69 to 0.90; *I*^2^ = 8%, *P* = 0.0005). Only one RCT described NIV+APP vs. NIV alone in the subgroup analysis and showed no difference in intubation rate (7/12 vs. 4/20). Although there was no significant statistics heterogeneity, we conducted subgroup analyses based on the predefined influence factors. In general, a significant reduction in the risk of intubation was also observed if only RCTs from populations with the following characteristics were pooled, which included sample size ≥200, HFNC/NIV % ≥70%, mean SpO_2_/FiO_2_ <150 mmHg, or more obese (≥40%) patients, or longer duration of APP (>8 hours), or age ≥70 years old, or mortality ≥20% of the population (Table 3). As for the analysis of observational studies, pooled estimates also showed that APP was significantly associated with a reduced risk of intubation (Supplementary material 9).

**Table 3:** Subgroup analyses of the outcome of intubation rate.

**Subgroup**		**References**	**N**	**RR/MD (95 % CI)**	**I^2^**	***P***
**Intubation rate**						
HFNC/NIV, %	>70	(14–16, 22–26)	1,596	0.83 [0.73, 0.95]	0	0.006
	≤70	(11, 12)	90	1.00 [0.38, 2.61]	0	1.0
Sample size	≥200	(14, 15, 23, 24)	1,454	0.84 [0.73, 0.96]	7	0.01
	<200	(11, 12, 16, 22, 25, 26)	232	0.91 [-1.27,3.10]	0	0.36
SpO_2_/FiO_2_	≥150	(16, 22–26)	781	0.91 [0.75, 1.10]	0	0.33
	<150	(14, 15)	905	0.78 [0.66, 0.93]	0	0.006
Obesity, %	<40	(11, 12, 14, 16, 24, 26)	980	0.90 [0.76, 1.07]	0	0.12
	≥40	(15, 22, 23, 25)	706	0.75 [0.62, 0.92]	9	0.006
Mean daily APP duration	<8 h	(11, 12, 14, 22–26)	1,181	0.88 [0.76, 1.03]	0	0.84
	≥8 h	(15, 16)	505	0.74 [0.58, 0.93]	3	0.01
Mortality prevalence	<20%	(12, 16, 22, 24, 25)	591	0.92 [0.72, 1.12]	0	0.34
	≥20%	(11, 14, 15, 23, 26)	1,095	0.80 [0.69, 0.94]	0	0.008
Age, years	≥60	(16, 22–24, 26)	766	0.94 [0.77, 1.13]	0	0.49
	<60	(11, 12, 14, 15, 25)	920	0.76 [0.64, 0.91]	0	0.002

APP, awake prone positioning; HFNC, high flow nasal cannula; ICU, intensive care unit; MD, mean difference; N, number of patients; NIV, non-invasive ventilation; RR, ratio risk; S/F, ratio of pulse oxygen saturation to fraction of inhaled oxygen; SpO_2_, pulse oxygen saturation.

### Secondary outcomes

**Figure 4:**
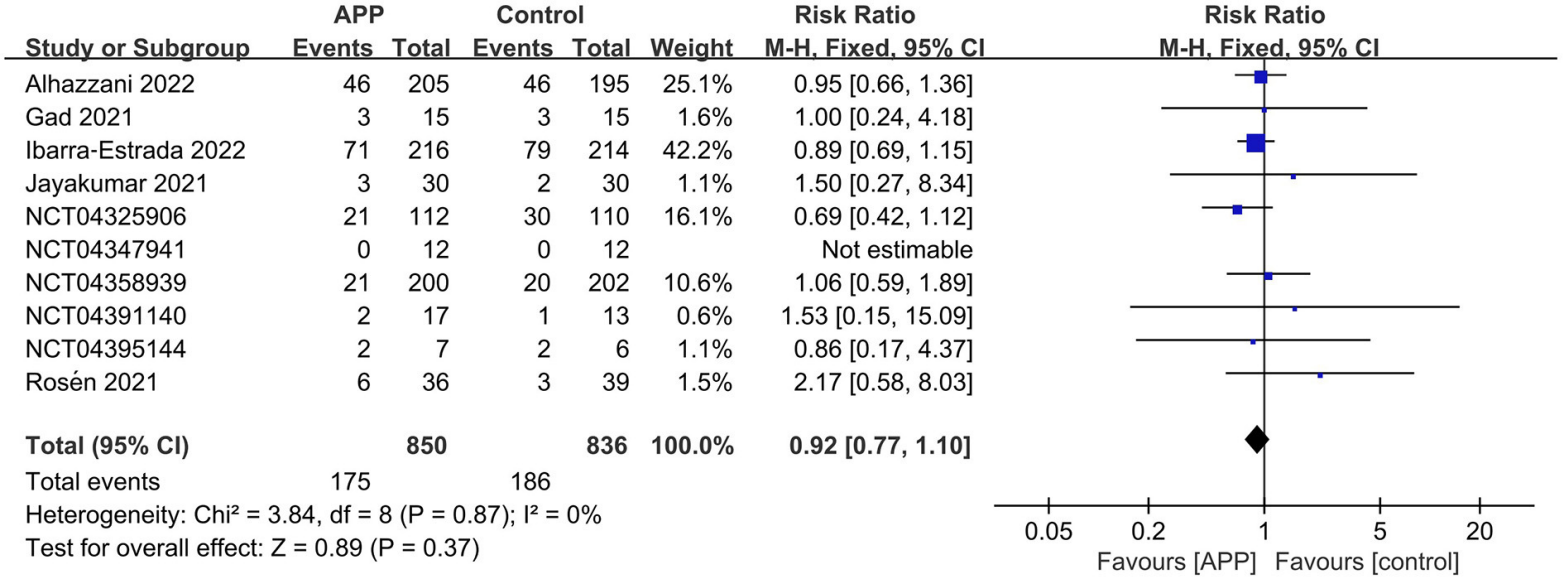
Forest plots of the awake prone position on mortality rates in COVID-19 patients in the intensive care unit.

**Figure 5:**
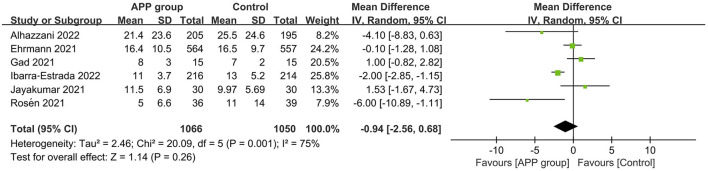
Forest plots of the effects of awake prone position on the length of stay in the intensive care unit.

A total of 10 RCTs reported the outcome of all-cause mortality, of which 7 described the 28-day mortality (15, 16, 22–26), and the other three provided 60-day mortality (14), hospital mortality (11), and ICU mortality rate (12), respectively. The pooled estimates suggested that APP did not significantly reduce mortality (10 RCTs, *n* = 1,686; RR 0.92; 95% CI, 0.77–1.10; *I*^2^ = 0%; *P* = 0.37. Figure 4). Moreover, there were no significant differences in the subgroup analyses, including types of sample size, HFNC/NIV percentage, SpO_2_/FiO_2_ level, obesity percentage, daily APP duration, age, or mortality prevalence (Supplementary material 10). The outcome of ICU LOS was available in 5 trials; no significant difference was found between the APP and control groups (5 RCTs, *n* = 1,686; MD = −0.58 days; 95% CI, −2.49 to 1.32; *I*^2^ = 63%; *P* = 0.55) (10–12, 14, 16) (Figure 5), regardless of the subgroups of all the predefined factors (Supplementary material 10). A total of four trials reported the AEs, which are present in Supplementary material 11. Only three RCTs provided oxygenation data with different oxygenation parameters (10–12). God et al. found that mean SaO2 significantly increased in both APP (from 79 ± 8.5% to 93 ± 5.9%) and NIV (from 827.1% to 95 ± 4.2%) groups (11). Jayakumar et al. found APP group patients (198.5 ± 87.6 mmHg) had a higher mean PaO_2_/FiO_2_ than the control group (171.7 ± 100.6 mmHg) without statistical differences (*P* = 0.3) (12). In the RCTs by Ehrmann et al., the authors reported that SpO_2_:FiO_2_, respiratory rate, and ROX index significantly improved during the first APP session, which lasted a median of 3 h, and this improvement persisted after returning to the supine position (10). Overall, the incidence of AEs associated with APP was extremely low, with each type of adverse reaction reported by a maximum of 2 RCTs. When pooled, we did not find differences between groups for these AEs, including catheter dislodgement (RR 0.92; 95CI% 0.77, 1.10) (10, 14) and skin breakdown (RR 0.52; 95CI%, 0.24, 1.14) (10, 16). As to the analyses in observational studies, APP significantly reduced mortality but showed no differences in ICU LOS and all AEs compared with control. Details were described in summarized in Supplementary materials 12, 13.

## Discussion

The current systematic review and meta-analysis of 10 RCTs (11, 12, 14–16, 22–26) and 12 observational studies (total N = 3,569) suggested that APP was associated with a significant reduction in the risk of intubation compared with the supine position in patients with COVID-19 pneumonia. APP did not significantly reduce the all-cause mortality and ICU LOS. These different outcomes in the APP group were further confirmed in sensitivity and subgroup analyses. In addition, APP was safe and showed no difference in AEs compared to control.

In the present meta-analysis, we included only all published RCTs and recruited only COVID-19 patients in the ICU setting. Thus, inconsistent findings and high heterogeneity of previous meta-analyses (6–9, 28–30) were addressed. Moreover, we included each of the six independently registered RCTs (22–27) in that recent multinational multicenter meta-trial (10) in our meta-analysis. This allowed the six trials to retain their study characteristics and made it possible to provide sufficient statistical efficacy to perform subgroup analyses to explore clinically influences of concern further. Fortunately, our main results appear robust, without heterogeneity, and validate several potential APP clinical influences. Moreover, our results provide some of the evidence needed for the 2019 Sepsis Survival Campaign Guidelines recommendations, which state that there is insufficient evidence to issue a recommendation on the use of APP in non-intubated adults with severe COVID-19.

The reduced intubation risk is relevant to the improved oxygenation, which can be explained as follows. First, the application of APP to COVID-19 pneumonia comes from previous experience with ARDS patients receiving the PP (2). Thus, the improved oxygenation can be explained by similar pathophysiological mechanisms, including a reduction in ventilation/perfusion mismatch, hypoxemia, shunt, and a more homogeneous lung area distribution (31). Our subgroup analyses suggest that patients with lower SaO_2_/FiO_2_, high-level respiratory supports (e.g., HFNC or NIV), and a higher mortality risk might benefit more from APP. These results are similar to previous studies confirming that PP can benefit severe ARDS patients rather than those with mild ARDS patients (2). Second, most patients (88.7%, 1,495/1,686) received HFNV and NIV with positive pressure, increasing end-expiratory lung volume and more uniform distribution of lung ventilation (32). Meanwhile, APP may reduce respiratory effort, thereby decreasing the incidence of self-induced lung injury. In addition, APP is safe. Previous studies have shown that a PP combined with invasive mechanical ventilation may increase the risk of pressure sores, tracheal tube dislocation or obstruction, drainage tube dislocation, and venous access removal (1, 33). However, we found the risks associated with APP are extremely low in almost negligible numbers.

Our results suggest that APP did not affect mortality. It may be related to the following reasons. First, the actual APP duration of the recruited patients is insufficient (2, 11, 12, 14, 22–26). The persistence of improved oxygenation was limited when patients returned from prone to a supine position (34, 35). In our study, most awake patients could not tolerate prolonged PP, whereas previous guidelines recommended 12 to 16 h of treatment duration based on data from adequately sedated ARDS patients (2, 36). Second, the average mortality rate of the included patients was less than 20%, which may not be easy to yield positive results. Third, the different clinical experiences of each center, APP implementation protocol, disease severity, and medical resources might affect patient prognosis. Fourth, the benefits of APP may be somewhat be offset, such as advanced non-invasive respiratory supports applied in both groups of patients (10, 16) or the cross-over phenomenon in control group patients who also spent some hours in the PP (12, 16). As reported by Jayakumar et al., 53% (16/30) of the control patients also received APP treatment (12). Interestingly, a small RCT (*n* = 75) explained their early trial termination due to the increased duration of APP in the control patients (mean 3.4 h) (16). Five, APP may be more beneficial for COVID-19 individuals who have CT evidence of early alveolar consolidation. However, some hypoxemic respiratory failure in COVID-19 individuals was caused by early pulmonary vascular damage and thrombosis without extensive alveolar consolidation. This might explain why the groups have similar morals. Finally, different phenotypes of COVID-19 may show different responses to APP. As demonstrated by Ibarra-Estrada et al., silent hypoxemic patients (defined as those with SpO_2_ < 90% in ambient air and no perceived dyspnea or shortness of breath) had fewer deaths compared to hypoxemic patients with dyspnea (23 vs. 39%, *P* = 0.001) when receiving APP (15).

The above evidence suggests that the efficacy of APP appears to relate to its duration. However, the optimal duration of the APP still needs further validation. Ibarra-Estrada et al. found that APP patients with a period of >8 h/day during the first 3 days had higher 28-day intubation-free survival compared to those with a period of <8 hours/day (114/122 [93%] vs. 14/94 [14%], *P* < 0.001) (15). Moreover, longer APP duration was significantly correlated with an adjusted risk of treatment success (r = 0.70) (15). The mean actual APP duration in the current meta-analysis ranged from <6 to 9 h daily, much lower than the targets set by each included study (11, 12, 14–16, 22–26). In several included RCTs that explicitly prescribed APP targets, Jayakumar et al. reported only 43% could adhere to their protocol which required a cumulative 6 h daily of APP (12). A similar result was also seen in a study that targeted an APP duration of 16 h, with only 6% of patients could achieve this goal, and the average daily duration was only 9 h (16). Moreover, Alhazzani et al. found that 85% of the APP group patients achieved the target duration on the first day, decreasing to 58% after 3 days (14). This suggests that adherence may be a potential limitation to APP efficacy. The picture is even less promising for studies that did not specify a protocol of APP duration. In that multi-national meta-RCT (10) conducted in 6 countries, the aim was to apply daily APP for as long as patients tolerated it. However, the actual mean APP duration in 5 of these countries was 1.7–3.1 h daily (22–26).

One issue to be addressed is how to improve adherence to the APP protocol. Given the low incidence and the mild symptoms, AEs may not be the main reason for poor compliance. Of note, 42% of the included patients were obese, which could be a potential limitation. Obese patients are more likely to benefit from APP by reducing chest wall weight and thus reducing respiratory work (37, 38). Therefore, more attention needs to be paid to this population. In addition, Alhazzani et al. summarized the poor adherence reasons and found that patient preference was 71% of the APP group, while care team preference was 55% of the control group (14). These results showed that adequate communication between the care team and the patient is critical, and the patient should fully understand the APP procedure and cooperate with it (39). Some methods included optimized analgesia, prior education (39), alternating PP (40), and assistive devices to improve patient comfort (41). Some new support devices (i.e., postural aids or mattresses) have also been developed to reduce discomfort, and evaluating their impact on adherence to the APP protocol is required.

In addition, APP is not utterly risk-free for patients with COVID-19. In addition to its contraindications related to the position, APP may overlook some patients at potential risk of disease deterioration. The risks are associated with poor ability to monitor respiratory function, such as respiratory rate and respiratory effort, especially in silent hypoxemic COVID-19 patients during APP (15). This can lead to delays in intubation and invasive ventilation. Therefore, further identification of subgroups that cannot benefit, based on the results of the subgroup analysis in our article, is necessary.

Our study has several limitations. First, due to resource factors, some severe COVID-19 patients who had to receive APP outside of ICU during the outbreak could not be enrolled in our study, leading to potential publication bias. Second, the recommended treatment strategies associated with COVID-19 pneumonia (e.g., remdesivir, traditional Chinese medicine, glucocorticoids, or hydroxychloroquine) changed over the period covered by the included RCTs, thus influencing clinical decisions and outcomes. Third, our subgroup-analysis results need to be interpreted with caution due to the small number of included trials. Similarly, five independently registered RCTs (22–26) could not provide sufficient information, including some secondary outcomes. Fourth, despite the subgroup analyses, the heterogeneity analysis of all these studies may be insufficient. Five, the unblind performance of APP may show a high risk of bias. Finally, studies targeting prolonged APP duration (>12 h or longer) are insufficient (16), so more research is still needed to determine whether prolonged APP reduces mortality in critical COVID-19 patients.

## Conclusion

Based on current evidence, APP is safe and significantly associated with reducing intubation rates in ICU patients with COVID-19 pneumonia, although its effectiveness is compromised due to limitations in patient's poor compliance. The efficacy of APP in reducing intubation seems to correlate with its duration. In addition, ICU COVID-19 patients with more serious (i.e., requiring HFNC/NIV, mean SpO_2_/FiO_2_ < 150 mmHg, or higher mortality risk), obese, or age ≥70 years old are more likely to benefit from APP. Therefore, well-designed RCTs with good patient adherence and further identifying the subgroup of patients most likely to benefit from this strategy are needed in the future.

### Take-home message

Ten Randomized controlled trials with a combined population of 1,686 non-intubation COVID-19 patients in the ICU setting were included in a meta-analysis. The use of awake prone positioning was associated with a significant reduction in intubation risk to the supine position (32.2 vs. 38.5%, *P* = 0.007).

Awake prone positioning did not affect the outcomes of mortality and length of stay in ICU in such a patient population.

## Data availability statement

The original contributions presented in the study are included in the article/Supplementary material, further inquiries can be directed to the corresponding author.

## Author contributions

H-BH and Y-BZ searched the scientific literature and drafted the manuscript. YY helped to collect the data and performed statistical analyses. BD contributed to the conception, design, data interpretation, manuscript revision for critical intellectual content, and study supervision. All authors read and approved the manuscript.

## Funding

This study was supported by Tsinghua University Spring Breeze Fund (2021Z99CFY019) and the Science and Technological Innovation Project of China Academy of Chinese Medical Sciences (CACMS) Innovation Fund (CI2021A02904).

## Conflict of interest

The authors declare that the research was conducted in the absence of any commercial or financial relationships that could be construed as a potential conflict of interest.

## Publisher's note

All claims expressed in this article are solely those of the authors and do not necessarily represent those of their affiliated organizations, or those of the publisher, the editors and the reviewers. Any product that may be evaluated in this article, or claim that may be made by its manufacturer, is not guaranteed or endorsed by the publisher.

## Abbreviations

APP: awake prone positioning
CI: confidence interval
COVID-19: Corona Virus Disease 2019
HFNC: high-flow nasal cannula
ICU: intensive care unit
MD: mean difference
MV: mechanical ventilation
NIV: non-invasive ventilation
RR: risk ratio
RCTs: randomized controlled trials
SD: standard deviations.
