# Supplementary material for: Awake prone positioning for patients with COVID-19 pneumonia in intensive care unit: A systematic review and meta-analysis
Source: Front Med (Lausanne). 2022 Sep 9;9:984446. doi: 10.3389/fmed.2022.984446 (PMC9500207; doi:10.3389/fmed.2022.984446)
Supplement: Supplementary file 1 [file Data_Sheet_1.docx]

**Awake Prone Positioning for COVID-19 requiring Intensive Care Unit Admission: A systematic review and meta-analysis**

Hui-Bin Huang MD; Yan Yao, MD; Yi-Bing Zhu, MD; Bin Du*, MD

*Corresponding author: Bin Du, Email: dubin98@gmail.com.

**Additional files**

Additional file 1 PRISMA checklist……………………………………………………………………………………….………………………..2

Additional file 2 Search Strategy………………………………………………………………………….………………………………………..5

Additional file 3 Studies needed for full-reviewed but not included ……………………………………………………...….....6

Additional file 4 Characteristics of included observational studies………………………………………………………………..8

Additional file 5 Quality assessment and overall risk of bias of included observational studies……..……………..9

Additional file 6 Funnel plot of comparison in RCTs: intubation rate……………………………………….………..…………..11

Additional file 7 Funnel plot of comparison in RCTs: intubation rate……………………………………….………..…………..12

Additional file 8 Forest plots of the intubation rate in the analysis of observational studies…………………………..13

Additional file 9 Subgroup analyses of the secondary outcomes………………………………………………..………………..14

Additional file 10 Adverse events across the RCTs…..…………….…………………………………………………………….……….15

Additional file 11 Secondary outcomes in the analysis of observational studies…………………………….…………….16

Additional file 12 Adverse events across the observational studies…..………………..….…………………………………….17

**Additional file 1 PRISMA 2009 checklist**

| **Section/topic** | **#** | **Checklist item** | **Reported on page #** |
| --- | --- | --- | --- |
| **TITLE** | | |  |
| Title | 1 | Identify the report as a systematic review, meta-analysis, or both. | 1 |
| **ABSTRACT** | | |  |
| Structured summary | 2 | Provide a structured summary including, as applicable: background; objectives; data sources; study eligibility criteria, participants, and interventions; study appraisal and synthesis methods; results; limitations; conclusions and implications of key findings; systematic review registration number. | 2-3 |
| **INTRODUCTION** | | |  |
| Rationale | 3 | Describe the rationale for the review in the context of what is already known. | 5-6 |
| Objectives | 4 | Provide an explicit statement of questions being addressed with reference to participants, interventions, comparisons, outcomes, and study design (PICOS). | 6 |
| **METHODS** | | |  |
| Protocol and registration | 5 | Indicate if a review protocol exists, if and where it can be accessed (e.g., Web address), and, if available, provide registration information including registration number. | 7 |
| Eligibility criteria | 6 | Specify study characteristics (e.g., PICOS, length of follow-up) and report characteristics (e.g., years considered, language, publication status) used as criteria for eligibility, giving rationale. | 7 |
| Information sources | 7 | Describe all information sources (e.g., databases with dates of coverage, contact with study authors to identify additional studies) in the search and date last searched. | 6 |
| Search | 8 | Present full electronic search strategy for at least one database, including any limits used, such that it could be repeated. | 7 and Additional file 2 |
| Study selection | 9 | State the process for selecting studies (i.e., screening, eligibility, included in systematic review, and, if applicable, included in the meta-analysis). | 7, Figure 1  And Additional file 3 |
| Data collection process | 10 | Describe method of data extraction from reports (e.g., piloted forms, independently, in duplicate) and any processes for obtaining and confirming data from investigators. | 8 |
| Data items | 11 | List and define all variables for which data were sought (e.g., PICOS, funding sources) and any assumptions and simplifications made. | 8 |
| Risk of bias in individual studies | 12 | Describe methods used for assessing risk of bias of individual studies (including specification of whether this was done at the study or outcome level), and how this information is to be used in any data synthesis. | 8 |
| Summary measures | 13 | State the principal summary measures (e.g., risk ratio, difference in means). | 8 |
| Synthesis of results | 14 | Describe the methods of handling data and combining results of studies, if done, including measures of consistency (e.g., I^2^) for each meta-analysis. | 9 |

| Risk of bias across studies | 15 | Specify any assessment of risk of bias that may affect the cumulative evidence (e.g., publication bias, selective reporting within studies). | 9 |
| --- | --- | --- | --- |
| Additional analyses | 16 | Describe methods of additional analyses (e.g., sensitivity or subgroup analyses, meta-regression), if done, indicating which were pre-specified. | 9 |
| **RESULTS** | | |  |
| Study selection | 17 | Give numbers of studies screened, assessed for eligibility, and included in the review, with reasons for exclusions at each stage, ideally with a flow diagram. | 11 |
| Study characteristics | 18 | For each study, present characteristics for which data were extracted (e.g., study size, PICOS, follow-up period) and provide the citations. | 11, Table 1-2, Additional file 4 |
| Risk of bias within studies | 19 | Present data on risk of bias of each study and, if available, any outcome level assessment (see item 12). | Additional file 5-6 |
| Results of individual studies | 20 | For all outcomes considered (benefits or harms), present, for each study: (a) simple summary data for each intervention group (b) effect estimates and confidence intervals, ideally with a forest plot. | 11-12 |
| Synthesis of results | 21 | Present results of each meta-analysis done, including confidence intervals and measures of consistency. | 12-13 |
| Risk of bias across studies | 22 | Present results of any assessment of risk of bias across studies (see Item 15). | 12-13 |
| Additional analysis | 23 | Give results of additional analyses, if done (e.g., sensitivity or subgroup analyses, meta-regression [see Item 16]). | 12-14 |
| **DISCUSSION** | | |  |
| Summary of evidence | 24 | Summarize the main findings including the strength of evidence for each main outcome; consider their relevance to key groups (e.g., healthcare providers, users, and policy makers). | 15-19 |
| Limitations | 25 | Discuss limitations at study and outcome level (e.g., risk of bias), and at review-level (e.g., incomplete retrieval of identified research, reporting bias). | 19 |
| Conclusions | 26 | Provide a general interpretation of the results in the context of other evidence, and implications for future research. | 17 |
| **FUNDING** | | |  |
| Funding | 27 | Describe sources of funding for the systematic review and other support (e.g., supply of data); role of funders for the systematic review. | 20 |

**Additional file 2: Search Strategy**

Search completed 25th May 2022

((("COVID-19"[Mesh]) OR (((((((((((((((((((((((((((((((((((((SARS-CoV-2 Infection) OR (Infection, SARS-CoV-2)) OR (SARS CoV 2 Infection)) OR (SARS-CoV-2 Infections)) OR (2019 Novel Coronavirus Disease)) OR (2019 Novel Coronavirus Infection)) OR (2019-nCoV Disease)) OR (2019 nCoV Disease)) OR (2019-nCoV Diseases)) OR (Disease, 2019-nCoV)) OR (COVID-19 Virus Infection)) OR (COVID 19 Virus Infection)) OR (COVID-19 Virus Infections)) OR (Infection, COVID-19 Virus)) OR (Virus Infection, COVID-19)) OR (Coronavirus Disease 2019)) OR (Disease 2019, Coronavirus)) OR (Coronavirus Disease-19)) OR (Coronavirus Disease 19)) OR (Severe Acute Respiratory Syndrome Coronavirus 2 Infection)) OR (SARS Coronavirus 2 Infection)) OR (COVID-19 Virus Disease)) OR (COVID 19 Virus Disease)) OR (COVID-19 Virus Diseases)) OR (Disease, COVID-19 Virus)) OR (Virus Disease, COVID-19)) OR (2019-nCoV Infection)) OR (2019 nCoV Infection)) OR (2019-nCoV Infections)) OR (Infection, 2019-nCoV)) OR (COVID19)) OR (COVID-19 Pandemic)) OR (COVID 19 Pandemic)) OR (Pandemic, COVID-19)) OR (COVID-19 Pandemics)) OR (COVID 19)) OR (COVID-19))) AND (((((prone position[Title/Abstract]) OR (prone positioning[Title/Abstract])) OR (proning[Title/Abstract])) OR (pronation[Title/Abstract])) OR ("Prone Position"[Mesh]))) AND (("Critical Care"[Mesh]) OR ((("Critical Care"[Mesh]) OR ((((critical care[Title/Abstract]) OR (critically ill[Title/Abstract])) OR (intensive care[Title/Abstract])) OR (((((((((((((((Critical Illness[Title/Abstract]) OR (Critical Care[Title/Abstract])) OR (intensive care units[Title/Abstract])) OR (Burn units[Title/Abstract])) OR (coronary care units[Title/Abstract])) OR (respiration, artificial[Title/Abstract])) ) OR (ventilators, mechanical[Title/Abstract])) OR (pulmonary ventilation[Title/Abstract])) OR (respiratory insufficiency[Title/Abstract])) OR (multiple organ failure[Title/Abstract])) OR (systemic inflammatory response syndrome[Title/Abstract])) OR (respiratory distress syndrome, adult[Title/Abstract])) OR (sepsis[Title/Abstract])) OR (shock, septic[Title/Abstract]))))))

**Additional file 3:** **Studies needed for full-reviewed but not included in the current meta-analysis (n=23 trials).**

| No | Study | Reason of exclusion |
| --- | --- | --- |
| 1 | Numata K, Sato K, Fujitani S, Kobayashi D. Respiratory Failure in COVID-19 with Awake Prone Positioning and HFNC Therapy: Aggravating Factors. Disaster Med Public Health Prep. 2021 Sep 22:1-3. | Inappropriate control |
| 2 | Padrão EMH, Rahhal H, Valente FS, Besen BAMP. Methodological issues in meta-analyses of observational studies: the need for attention to the details. Br J Anaesth. 2022 May;128(5):e303-e305. | Irrelevant to the current study |
| 3 | Ding L, Wang L, Ma W, He H. Efficacy and safety of early prone positioning combined with HFNC or NIV in moderate to severe ARDS: a multi-center prospective cohort study. Crit Care. 2020 Jan 30;24(1):28. | Inappropriate control |
| 4 | Qian ET, Gatto CL, Amusina O, Dear ML, Hiser W, Buie R, et.al; Vanderbilt Learning Healthcare System Platform Investigators. Assessment of Awake Prone Positioning in Hospitalized Adults With COVID-19: A Nonrandomized Controlled Trial. JAMA Intern Med. 2022 Apr 18:e221070. | Reported without ICU admission data |
| 5 | Taboada M, Baluja A, Santos LD, González I, Veiras S, et, al. Effectiveness of dexmedetomidine combined with high flow nasal oxygen and long periods of awake prone positioning in moderate or severe COVID-19 pneumonia. J Clin Anesth. 2021 Sep;72:110261. | Inappropriate control |
| 6 | Thompson AE, Ranard BL, Wei Y, Jelic S. Prone Positioning in Awake, Nonintubated Patients With COVID-19 Hypoxemic Respiratory Failure. JAMA Intern Med. 2020 Nov 1;180(11):1537-1539. doi: 10.1001/jamainternmed.2020.3030. PMID: 32584946; PMCID: PMC7301298. | Inappropriate control |
| 7 | Dubosh NM, Wong ML, Grossestreuer AV, Loo YK, Sanchez LD, Chiu D, Leventhal EL, Ilg A, Donnino MW. Early, awake proning in emergency department patients with COVID-19. Am J Emerg Med. 2021 Aug;46:640-645. | Reported without ICU admission |
| 8 | Khanum I, Samar F, Fatimah Y, Safia A, Adil A, Kiren H, Nasir N, Faisal M, Bushra J. Role of awake prone positioning in patients with moderate-to-severe COVID-19: an experience from a developing country. Monaldi Arch Chest Dis. 2021 Mar 5;91(2). | Reported without ICU admission |
| 9 | Aisa T, Hassan T, Khan E, Algrni K, Malik MA. Efficacy and feasibility of awake proning in patients with COVID-19-related acute hypoxemic respiratory failure: an observational, prospective study. Ir J Med Sci. 2022 Apr 14:1–5. | Reported without ICU admission |
| 10 | Tatlow C, Heywood S, Hodgson C, Cunningham G, Conron M, Ng HY, Georgiou H, Pound G. Physiotherapy-assisted prone or modified prone positioning in ward-based patients with COVID-19: a retrospective cohort study. Physiotherapy. 2022 Mar;114:47-53. | Reported without ICU admission |
| 11 | Padrão EMH, Valente FS, Besen BAMP, Rahhal H, Mesquita PS, et al. Awake Prone Positioning in COVID-19 Hypoxemic Respiratory Failure: Exploratory Findings in a Single-center Retrospective Cohort Study. Acad Emerg Med. 2020 Dec;27(12):1249-1259. | Reported without ICU admission |
| 12 | Dueñas-Castell C, Borre-Naranjo D, Rodelo D, Lora L, Almanza A, Coronell W, Rojas-Suarez J. Changes in Oxygenation and Clinical Outcomes with Awake Prone Positioning in Patients with Suspected COVID-19 In Low-Resource Settings: A Retrospective Cohort Study. J Intensive Care Med. 2021 Nov;36(11):1347-1353. | Reported without ICU admission |
| 13 | Chiumello D, Chiodaroli E, Coppola S, Cappio Borlino S, Granata C, Pitimada M, Wendel Garcia PD. Awake prone position reduces work of breathing in patients with COVID-19 ARDS supported by CPAP. Ann Intensive Care. 2021 Dec 20;11(1):179. | Inappropriate control |
| 14 | Coppo A, Bellani G, Winterton D, Di Pierro M, Soria A, Faverio P, Cairo M, Mori S, et al. Feasibility and physiological effects of prone positioning in non-intubated patients with acute respiratory failure due to COVID-19 (PRON-COVID): a prospective cohort study. Lancet Respir Med. 2020 Aug;8(8):765-774. | Reported without ICU admission |
| 15 | Jha A, Chen F, Mann S, Shah R, Abu-Youssef R, Pavey H, et al. Physiological effects and subjective tolerability of prone positioning in COVID-19 and healthy hypoxic challenge. ERJ Open Res. 2021 Feb 7;8(1):00524-2021. | Conducted in General ward |
| 16 | Perez-Nieto OR, Escarraman-Martinez D, Guerrero-Gutierrez MA, Zamarron-Lopez EI, Mancilla-Galindo J, et al. Awake prone positioning and oxygen therapy in patients with COVID-19: the APRONOX study. Eur Respir J. 2022 Feb 24;59(2):2100265. | Conducted in General ward |
| 17 | Zang X, Wang Q, Zhou H, Liu S, Xue X; COVID-19 Early Prone Position Study Group. Efficacy of early prone position for COVID-19 patients with severe hypoxia: a single-center prospective cohort study. Intensive Care Med. 2020 Oct;46(10):1927-1929. | Reported without ICU admission |
| 18 | Cammarota G, Rossi E, Vitali L, Simonte R, Sannipoli T, Anniciello F, Vetrugno L, Bignami E, Becattini C, Tesoro S, Azzolina D, Giacomucci A, Navalesi P, De Robertis E. Effect of awake prone position on diaphragmatic thickening fraction in patients assisted by noninvasive ventilation for hypoxemic acute respiratory failure related to novel coronavirus disease. Crit Care. 2021 Aug 24;25(1):305. | Without appropriate control |
| 19 | Sryma PB, Mittal S, Mohan A, Madan K, Tiwari P, Bhatnagar S, Trikha A, Dosi R, Bhopale S, Viswanath R, Hadda V, Guleria R, Baldwa B. Effect of proning in patients with COVID-19 acute hypoxemic respiratory failure receiving noninvasive oxygen therapy. Lung India. 2021 Mar;38(Supplement):S6-S10. | Reported without ICU admission |
| 20 | Althunayyan S, Almutary AM, Junaidallah MA, Heji AS, Almazroua F, Alsofayan YM, Al-Wathinani A, AlRuthia Y. Prone position protocol in awake COVID-19 patients: A prospective study in the emergency department. J Infect Public Health. 2022 Apr;15(4):480-485. | Conducted in the emergency department |
| 21 | Silva Junior JM, Treml RE, Golinelli PC, Segundo MRMG, Menezes PFL, et al. Response of patients with acute respiratory failure caused by COVID-19 to awake-prone position outside the intensive care unit based on pulmonary involvement. Clinics (Sao Paulo). 2021 Dec 10;76:e3368. | Reported without ICU admission |
| 22 | Chiumello D, Chiodaroli E, Coppola S, Cappio Borlino S, Granata C, Pitimada M, Wendel Garcia PD. Awake prone position reduces work of breathing in patients with COVID-19 ARDS supported by CPAP. Ann Intensive Care. 2021 Dec 20;11(1):179. |  |

**Additional file 4: Characteristics of included observational studies in the current meta-analysis and systemic review**

| Study | Country | N | Design | Usual care | Age, year | Male, % | BMI, kg/m^2^ | Mortality, % | NOS |
| --- | --- | --- | --- | --- | --- | --- | --- | --- | --- |
| Altinay 2022 | Turkey | 58 | SC, R | NRM | 62.4/72.6 | 44/39.1 | 25.1/26.6 | 52.1 | 7 |
| Barker 2021 | UK | 20 | SC, R | NIV | 59/64 | 60/60 | - | 25.0 | 7 |
| Esperatti 2022 | Argentina | 335 | MC, P | LF, VM, NRM | 57/66.5 | 76/78 | 30/30 | 20.3 | 8 |
| Ferrando 2020 | Spain | 199 | MC, P | HFNC | 60/63 | 75.9/72.7 | 26.8/27.3 | 12.6 | 8 |
| Jouffroy 2021 | France | 379 | MC, R | NIV/HFNC/CPAP | 59.5/62 | 90/75.2 | 28.5/28 | 26.4 | 7 |
| Meredith 2021 | USA | 113 | SC, R | NC, HFNC, NIV | 56.8/64.3 | 53.8/53.8 | 32.5/31.3 | 12.0 | 7 |
| Ni 2020 | China | 55 | SC, P | NC/FM/HFNC/NIV | 60/64 | 64.7/60 | - | - | 9 |
| Numata 2022 | Japan | 108 | MC, R | COT/HFNC | 68/70 | 68.5/42.6 | 23.9/24.2 | - | 7 |
| Pierucci 2021 | Italy | 32 | SC, P | HFNC/NIV | 59/70 | 81/62 | - | 9.4 | 7 |
| Simioli 2021 | Italy | 29 | SC, R | HFNC/CPAP | 61/71 | - | 28/28 | 10.3 | 8 |
| Tonelli 2021 | Italy | 114 | MC, R | HFNC/NIV | 61/70 | 66/73 | 26/28 | 19.3 | 6 |
| Vianello 2021 | Italy | 93 | SC, P | HFNC | 67/69 | 66/60 | 26.9/28.3 | 9.7 | 8 |

BMI, body mass index; COT, conventional oxygen therapy; CPAP, continuous positive airway pressure; HFNC, high flow nasal cannula; HFNO, high-flow nasal oxygen therapy; ICU, intensive care unit; NC = nasal catheter; NIV, non-invasive ventilation; NRM, non-rebreather mask; NOS, Newcastle-Ottawa Scale.

**Additional file 5:** **Quality assessment and overall risk of bias of included observational studies**

| First author / year | Patient selection | | | | Comparability | Outcome | | | Risk of bias |
| --- | --- | --- | --- | --- | --- | --- | --- | --- | --- |
|  | Representation of the exposed cohort | Selection of the non-exposed cohort | Ascertainment of exposure | Outcome of  interest not  present at start | Comparability of cohorts on the basis of the design or analysis | Assessment  of outcome | Was follow-up long enough for outcomes to occur | Adequacy of follow up of cohorts |  |
| Altinay 2022 | ★ | ★ | ★ | ☆ | ★ | ★ | ★ | ★ | 7 |
| Barker 2021 | ★ | ★ | ★ | ☆ | ★ | ★ | ★ | ★ | 7 |
| Esperatti 2022 | ★ | ★ | ★ | ★ | ★ | ★ | ★ | ★ | 8 |
| Ferrando 2020 | ★ | ★ | ★ | ★ | ★ | ★ | ★ | ★ | 8 |
| Jouffroy 2021 | ★ | ★ | ★ | ☆ | ★ | ★ | ★ | ★ | 7 |
| Meredith 2021 | ★ | ★ | ★ | ☆ | ★ | ★ | ★ | ★ | 7 |
| Ni 2020 | ★ | ★ | ★ | ★ | ★★ | ★ | ★ | ★ | 9 |
| Numata 2022 | ★ | ★ | ★ | ☆ | ★ | ★ | ★ | ★ | 7 |
| Pierucci 2021 | ★ | ★ | ★ | ★ | ☆☆ | ★ | ★ | ★ | 7 |
| Simioli 2021 | ★ | ★ | ★ | ☆ | ★★ | ★ | ★ | ★ | 8 |
| Tonelli 2021 | ★ | ★ | ★ | ☆ | ☆ | ★ | ★ | ★ | 6 |
| Vianello 2021 | ★ | ★ | ★ | ★ | ★ | ★ | ★ | ★ | 8 |

**Abbreviations:** H=high quality; M=moderate quality; L= low quality.

**Note:** A study was given a maximum of one point in each item within the patient selection and outcome domains and given a maximum of two points for the Comparability domain with the following criteria:

1. **Representation of the exposed cohort**：Studies received 1 point if they recruited consecutive series of adult patients with blood phosphate concentration tested, or all included patients or did not miss a large number of patients.

2. **Selection of the non-exposed cohort**：Studies received 1 point if both groups of patients with or without hyperphosphatemia (defined by each author) were recruited from the same cohort.

3. **Ascertainment of exposure**: Studies received 1 point if they had been demonstrated to have abnormal blood phosphate concentration.

4. **Outcome of interest was not present at start of study**: Studies received points if they demonstrated the outcome of interest was not present at the start of the study.

5. **Comparability:** Studies received points if they controlled the disease severity (i.e., SOFA, SAPS3, ISS or APACHEII scores) (1 point); or any additional important factors such as age, gender or ethnicities, APP duration; comorbidities, or there were no significant differences between hyperphosphatemia and normal phosphate concentration (1 point).

6. **Assessment of outcome**: Studies received 1 point if they had independent blind assessment or record linkage.

7. **Was follow-up long enough for outcomes to occur**: Studies received 1 point if they follow up until at least either inpatient mortality or for 30 days or had adequate record linkage.

8. **Adequacy of follow up for cohorts**: Studies received 1 point if all recruited subjects were all followed up, or the number lost to follow-up was unlikely to introduce bias (≤10%).

**Additional file 6: Funnel plot of comparison in RCTs: intubation rate**


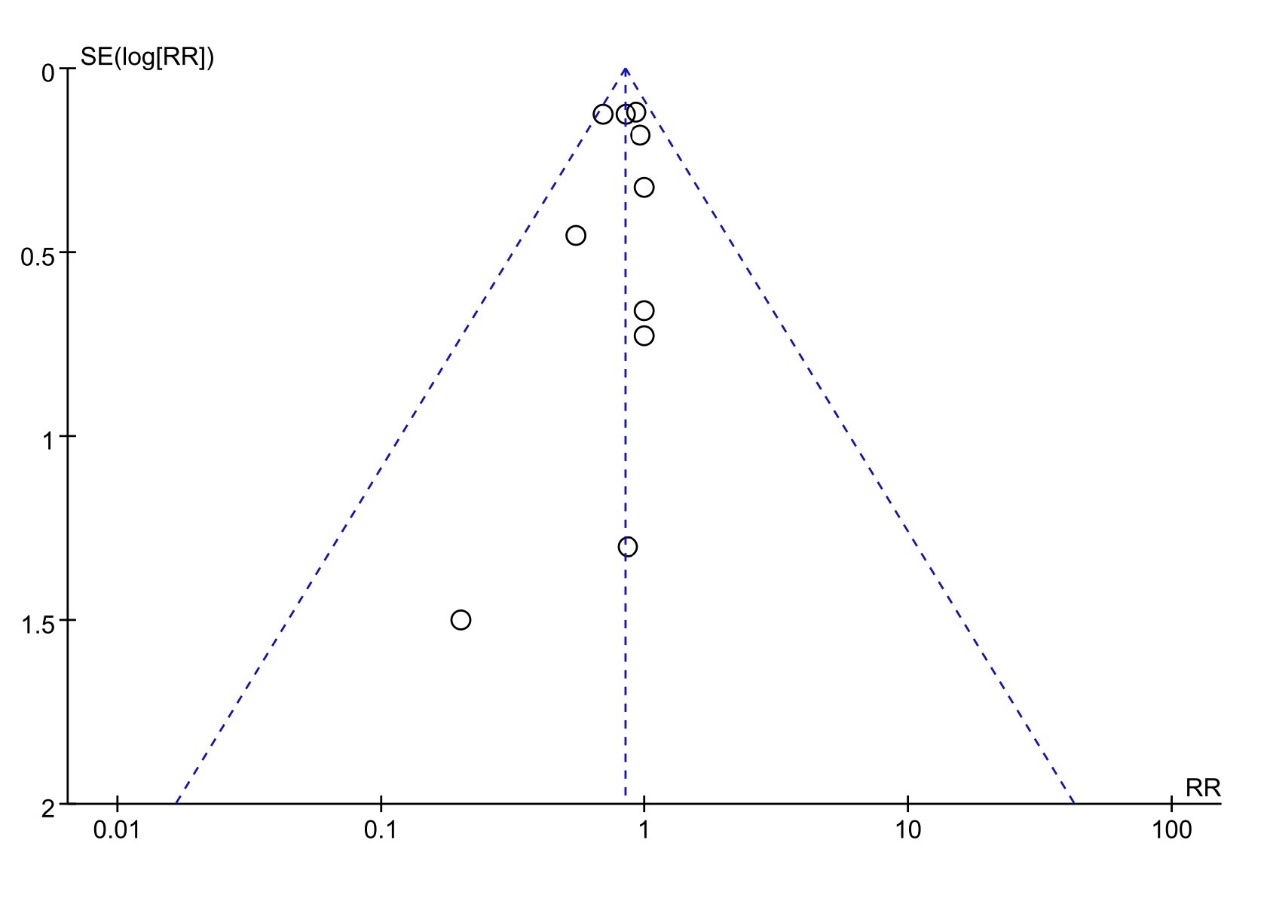


**Additional file 7: Summary of outcomes for the Effect of awake prone position on COVID-19 pneumonia**


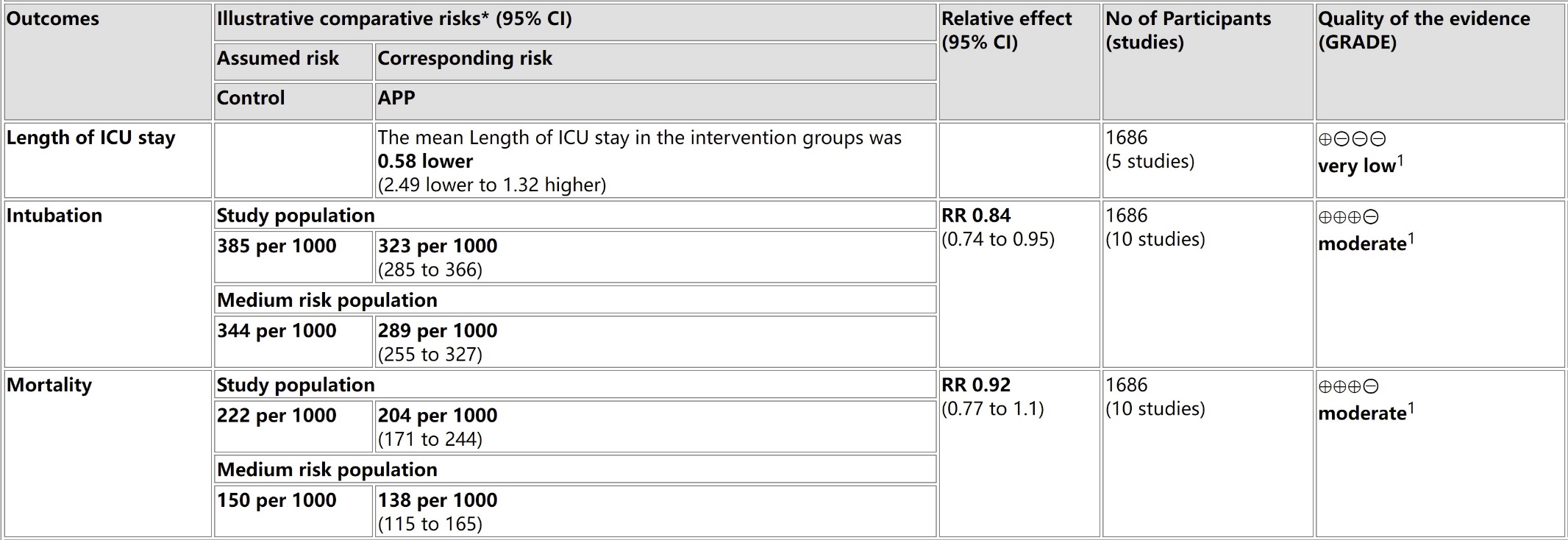


^1^GRADE Working Group grades of evidence

**High quality**: Further research is very unlikely to change our confidence in the estimate of effect.

**Moderate quality:** Further research is likely to have an important impact on our confidence in the estimate of effect and may change the estimate.

**Low quality**: Further research is very likely to have an important impact on our confidence in the estimate of effect and is likely to change the estimate.

**Very low quality**: We are very uncertain about the estimat

**Additional file 8: Forest plots of the intubation rate in the analysis of observational studies**


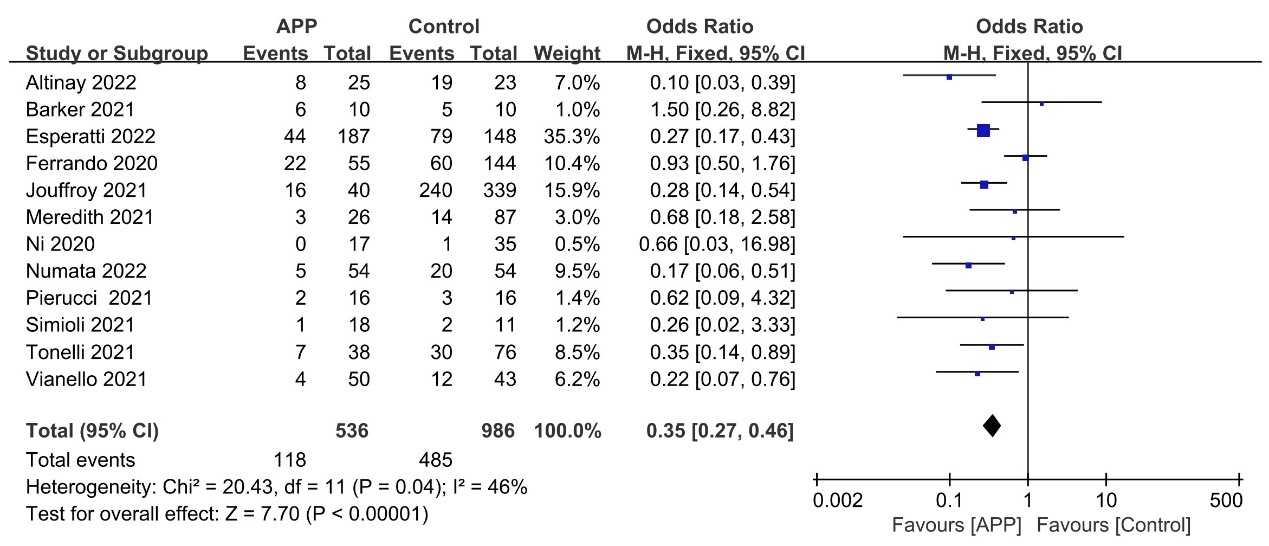


APP=awake prone position; CI = confidence interval.

**Additional file 9: Subgroup analyses of the secondary outcomes**

| Mortality | | | | | | |
| --- | --- | --- | --- | --- | --- | --- |
| HFNC/NIV, % | ≥70 | 14-16, 22-26 | 1597 | 0.92 [0.77, 1.10] | 0 | 0.34 |
|  | <70 | 11, 12 | 90 | 1.20 [0.40, 3.59] | 0 | 0.74 |
| Sample size | ≥200 | 14, 15, 23, 24 | 1454 | 0.89 [0.74, 1.07] | 0 | 0.22 |
|  | <200 | 11, 12, 16, 22, 25, 26 | 233 | 1.45 [0.71, 2.94] | 0 | 0.30 |
| SpO_2_/FiO_2_ | ≥150 | 16, 22-26 | 857 | 0.95 [0.68, 1.31] | 0 | 0.74 |
|  | <150 | 14, 15 | 830 | 0.91 [0.74 1.13] | 0 | 0.40 |
| Obesity, % | <40 | 11, 12, 14, 16, 24, 26 | 981 | 1.04 [0.79, 1.38] | 0 | 0.77 |
|  | ≥40 | 15, 22, 23, 25 | 706 | 0.90 [0.76, 1.07] | 0 | 0.14 |
| Mean daily APP duration | <8 h | 11, 12, 14, 22-26 | 1182 | 0.92 [0.71, 1.17] | 0 | 0.48 |
|  | ≥8 h | 15, 16 | 505 | 0.94 [0.73, 1.20] | 0 | 0.34 |
| Mortality prevalence | ＜20% | 12, 16, 22, 24, 25 | 591 | 1.24 [0.76, 2.02] | 0 | 0.39 |
|  | ≥20% | 11, 14, 15, 23, 26 | 1096 | 0.87 [0.72, 1.06] | 0 | 0.16 |
| Age, years | ≥60 | 16, 22-24, 26 | 736 | 0.91 [0.64, 1.28] | 6 | 0.58 |
|  | <60 | 11, 12, 14, 15, 25 | 950 | 0.93 [0.76, 1.14] | 0 | 0.48 |
| Length of stay in ICU | | | | | | |
| HFNC/NIV% | >70 | 10, 14, 16 | 1596 | -2.84 [-6.83, 1.16] | 73 | 0.16 |
|  | ≤70 | 11, 12 | 90 | 1.13 [-0.46, 2.72] | 0 | 0.16 |
| Sample size | ≥200 | 10, 14 | 1521 | -1.42 [-5.10, 2.27] | 61 | 0.45 |
|  | <200 | 11, 12, 16 | 165 | -0.54 [-4.04, 2.97] | 73 | 0.76 |
| SpO_2_/FiO_2_ | ≥150 | 12,16 | 135 | -2.00 [-9.36, 5.37] | 84 | 0.59 |
|  | <150 | 10, 14 | 1521 | -1.42 [-5.10, 2.27] | 61 | 0.45 |
| Obesity, % | <40 | 10, 11, 16 | 1226 | -0.58 [-2.88, 1.71] | 71 | 0.38 |
|  | ≥40 | 15 | 430 | -2.00 [-2.85, -1.15] | - | - |
| Mean daily APP duration | <8 h | 10-12,14 | 1611 | 0.17 [-0.76, 1.10] | 38 | 0.72 |
|  | ≥8 h | 15, 16 | 505 | -3.24 [-6.87, 0.39] | 60 | 0.08 |
| Mortality prevalence | ＜20% | 12, 16 | 135 | -2.00 [-9.36, 5.37] | 84 | 0.59 |
|  | ≥20% | 10, 11, 14, 16 | 1551 | -0.11 [-1.81, 1.58] | 51 | 0.90 |
| Age, years | ≥60 | 10, 16 | 1196 | -2.55 [-8.25, 3.15] | 81 | 0.38 |
|  | <60 | 11, 12, 14 ,15 | 920 | -0.66 [-2.92, 1.60] | 77 | 0.57 |

APP, awake prone positioning; HFNC, high flow nasal cannula; ICU = intensive care unit; MD = mean difference; N, number of patients; NIV, non-invasive ventilation; RR = ratio risk; S/F, ratio of pulse oxygen saturation to fraction of inhaled oxygen; SpO_2_, pulse oxygen saturation.

**Additional file 10: Adverse events across the RCTs**

|  | Alhazzani 2022  A vs. C | Ehrmann 2021  A vs. C | Jayakumar 2021  A vs. C | Rosén 2021  A vs. C |
| --- | --- | --- | --- | --- |
| Total | 21/205 vs. 0/195 |  | 0/30 vs. 0/30 |  |
| Pain or discomfort | 1/205 vs. 0/195 |  |  |  |
| Desaturation | 1/205 vs. 0/195 |  |  |  |
| Access removal/ dislodgement | 1/205 vs. 0/195 | 26/564 vs. 17/557 |  |  |
| Hypotension | 1/205 vs. 0/195 |  |  |  |
| Nausea/vomiting | 1/205 vs. 0/195 |  |  |  |
| Shortness of breath | 1/205 vs. 0/195 |  |  |  |
| Dizziness | 1/205 vs. 0/195 |  |  |  |
| Coughing | 1/205 vs. 0/195 |  |  |  |
| Skin breakdown |  | 8/564 vs. 10/557 |  | 2/36 vs. 9/39 |
| Vomiting |  | 15/564 vs. 18/557 |  |  |

A=awake prone position; C= control

**Additional file 11 Summarized the secondary outcomes in pooling observational studies**

**Forest plots of the APP on mortality rates compared with control.**


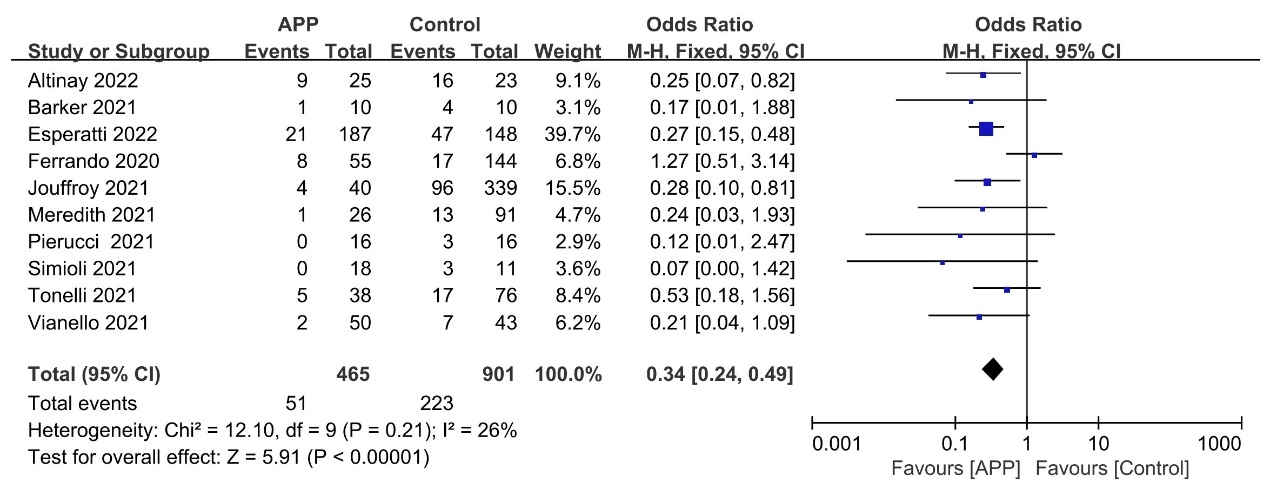


APP=awake prone position; CI = confidence interval.

**Forest plots of the APP on length of stay in ICU compared with control.**


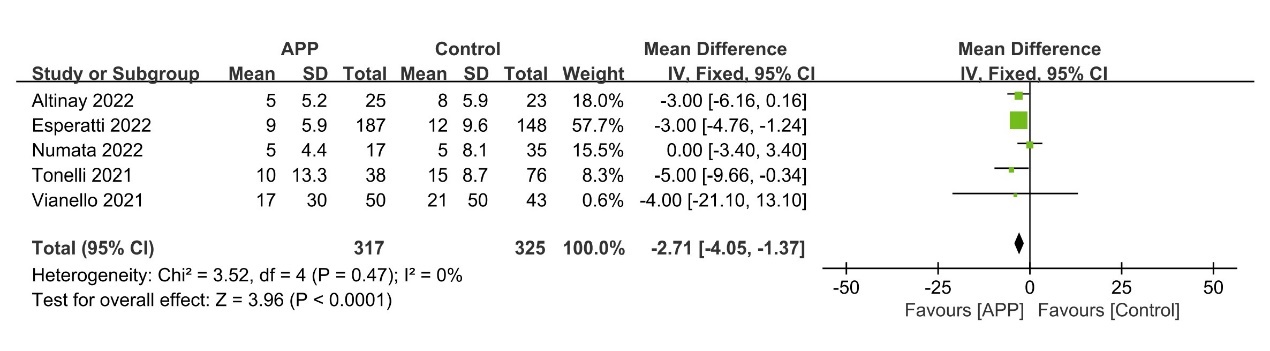


APP=awake prone position; CI = confidence interval.

**Additional file 12: Adverse events across the observational studies**

| Study | Description of adverse events in the included studies |
| --- | --- |
| Esperatti 2022 | Three patients had severe complications in the AW-PP group: syncope in the prone position requiring ETI, ardiorespiratory arrest requiring ETI, and arterial line displacement with bleeding and hypotension (not requiring ETI), respectively. |
| Ni 2020 | No adverse events occurred during the study. |
| Tonelli 2021 | We found that awake proning was safe, and no adverse events occurred in PP group. |
| Vianello 2021 | No adverse event was recorded during application of PP. |
